# A Machine Learning-Based Clinical Decision Support Tool for Intertrochanteric Hip Fracture Patients to Predict Postoperative Anemia Risk: A Retrospective Cohort Study

**DOI:** 10.3390/bioengineering13050489

**Published:** 2026-04-23

**Authors:** Xinbei Dong, Qinglong Wang, Zhipeng Huang, Yucai Wang

**Affiliations:** Department of Orthopedic Surgery, Tangdu Hospital, Fourth Military Medical University, No. 1 of Xinsi Road, Baqiao District, Xi’an 710038, China; 19567350216@163.com (X.D.); qinglongwang2019@163.com (Q.W.)

**Keywords:** intertrochanteric hip fracture, postoperative anemia, machine learning, prediction model, shapley additive explanation

## Abstract

Background: Postoperative anemia associated with intertrochanteric hip fracture is a detrimental complication that detrimentally impairs patients’ outcomes. This study is designed to develop an online predictive tool to assist physicians in developing surgical blood preparation strategies to prevent the occurrence of postoperative anemia. Methods: This study included data collected from June 2017 to June 2025 on intertrochanteric hip fracture patients at Tangdu Hospital, including demographic information, comorbidities, vital signs, and laboratory results. LASSO regression was used to select predictive variables, and seven machine learning techniques: Logistic Regression, Support Vector Machine, Decision Tree, LightGBM, XGBoost, Neural Networks, and Random Forest, were compared to identify the best tool for predicting postoperative anemia risk. We created a patient-specific risk prediction tool with SHAP-driven interpretability for clinical decision support. Results: A total of 815 patients were included in the analysis, of whom 208 (25.5%) presented with postoperative anemia. Eight variables were selected to build seven machine learning models. Among these, the SVM model exhibited the best predictive performance in terms of discrimination, calibration, and clinical applicability, with an AUC range of 0.827–0.831. In test sets encompassing diverse population characteristics, SVM achieved the highest sensitivity (72.73%), accuracy (77.78%), and F1 score (57.14%). Conclusions: We established an online prediction platform for clinical practice, enabling clinicians to assess anemia risk in intertrochanteric hip fracture patients and support early prevention of postoperative anemia.

## 1. Introduction

Intertrochanteric hip fractures represent a growing public health challenge, with incidence rising in parallel with aging populations and increased prevalence of osteoporotic bone disease [1]. These fractures are associated with substantial morbidity, mortality, and loss of independence, imposing significant clinical and socioeconomic burdens [2]. Surgical fixation, primarily with Proximal Femoral Nail Antirotation (PFNA), remains the standard of care; however, postoperative complications remain common.

Postoperative anemia is a frequent and clinically significant complication after intertrochanteric fracture surgery, with reported incidence rates up to 49.3% [3,4]. This condition contributes to delayed ambulation, prolonged hospitalization, increased transfusion requirements, and heightened risk of infections and cardiovascular events [5,6]. Current preventive strategies, including intraoperative blood transfusion and antifibrinolytic agents such as tranexamic acid [7], have demonstrated limited efficacy and are associated with inherent risks—including surgical site infections, transfusion-related adverse events, and immune reactions [8,9,10]. Consequently, the ability to stratify patients’ risk during the intraoperative period could enable targeted adjustments to postoperative care, optimizing resource utilization while ensuring patient safety.

Despite recognition of individual risk factors such as preoperative hemoglobin levels and surgical duration, existing literature lacks validated predictive models that integrate comprehensive preoperative and intraoperative variables for individualized risk stratification. Most prior studies [11,12,13] have been limited by small sample sizes and single-center designs, precluding generalizability. Furthermore, these studies have predominantly employed traditional statistical methods that are restricted to simple linear relationships and lack the capacity to capture complex nonlinear patterns inherent in clinical data. Such approaches also require manual variable selection and extraction, necessitating specialized domain knowledge and reliance on prior assumptions, thereby constraining model adaptability and scalability [14]. These limitations underscore the necessity of developing novel predictive methods capable of handling complex clinical data with adaptive learning capabilities.

Accordingly, the application of machine learning (ML) techniques provides an effective strategy to address these limitations, with advantages manifesting at three distinct levels [15]. At the algorithmic level, ML enables automatic learning of complex nonlinear relationships within high-dimensional data, substantially reducing reliance on manual feature engineering and rule-based programming [16]. At the model performance level, rigorously validated ML models demonstrate cross-dataset generalization potential, allowing learned patterns to be transferred to independent clinical cohorts while maintaining relatively stable predictive performance [17]. At the system integration level, modern computational frameworks support automated feature extraction and efficient processing of large-scale heterogeneous healthcare data, providing the technical foundation for rapid model deployment [18].

The objective of this study is to develop and validate a machine learning-based prediction tool for postoperative anemia in intertrochanteric hip fracture patients using a large cohort, thereby filling the current gap in individualized risk assessment tools, with the ultimate aim of predicting the risk of postoperative anemia and informing perioperative blood management strategies.

## 2. Methods

### 2.1. Data Source

The derivation set comprised consecutive patients with an intertrochanteric hip fracture who underwent proximal femoral nail antirotation (PFNA) fixation between June 2017 and December 2024. The temporal validation set included patients who underwent PFNA fixation between January and June 2025.

Eligibility criteria:

Inclusion: (1) Unilateral closed fracture; (2) PFNA fixation; (3) complete data on baseline and intraoperative variables.

Exclusion: (1) Polytrauma or multiple fractures; (2) Pathological fractures; (3) Preoperative hemoglobin < 80 g/L; (4) Missing primary outcome data.

The study enrolled 815 patients in total. A total of 761 patients (197 events, 25.9% anemia rate) constituted the derivation set (training and validation), and 54 patients formed the temporal test set.

This retrospective cohort study was performed at Tangdu Hospital. The study protocol was approved by the Ethics Committee of Tangdu Hospital. We reported the study in accordance with the TRIPOD guideline for prediction model development and validation.

### 2.2. Data Collection

#### 2.2.1. Outcome Definition

The primary outcome was postoperative anemia, defined as hemoglobin < 80 g/L within 7 days post-surgery [8]. Hemoglobin levels were measured routinely on postoperative days 1, 4, and 7 for all patients. This schedule was systematically executed by resident physicians and ward nurses as part of standard clinical care. If the patient received a blood transfusion within 7 days after surgery, the test values obtained before transfusion shall be used preferentially.

#### 2.2.2. Predictors

Based on a systematic literature review [19] and clinical expert consensus, we identified 15 candidate predictors: age, gender, BMI, Evans classification, type of anesthesia, operation time, preoperative waiting days, intraoperative transfusion volume, preoperative hemoglobin, platelet count, albumin, serum calcium, hypertension, diabetes, and osteoporosis.

#### 2.2.3. Missing Data Handling

Among 761 patients, 8 (1.05%) had missing data for 1–2 variables. Given the low missing rate, multiple imputation by chained equations (MICE) was performed with 20 imputations and 50 iterations, and estimates were pooled using Rubin’s rules. A complete-case sensitivity analysis was conducted for comparison.

### 2.3. Data Preprocessing

**Figure 1:**
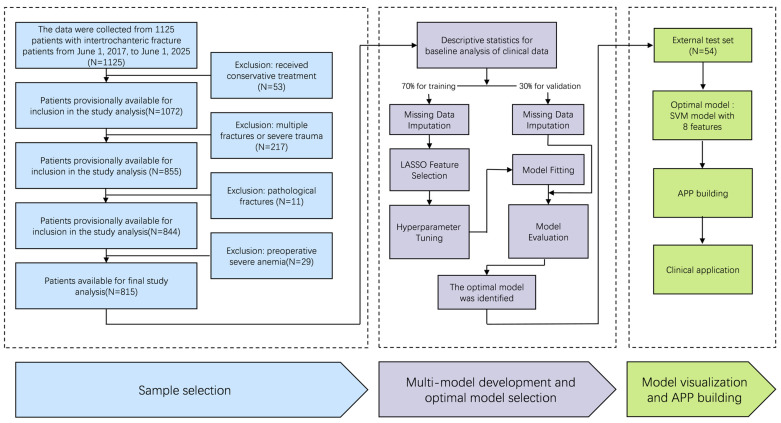
Flowchart of this study.

Figure 1 depicts the workflow of data preprocessing and model development. Briefly, the study proceeded through six sequential phases: data splitting, missing value imputation, feature selection, hyperparameter tuning, model fitting, and performance evaluation. Each phase is described in detail below. Predictor selection is crucial before model training, since it effectively alleviates overfitting and diminishes noise through the removal of irrelevant and redundant features [20]. To address missing data, we utilized multiple imputation, which helps minimize potential bias without excluding participants [21]. The dataset consists of 208 patients with anemia and 607 patients without anemia. To evaluate the generalization ability of the tool’s predictions and prevent overfitting, We randomly divided all patient data into a training set (70%) and a validation set (30%) [22], with data processing and model construction was performed on the training set. To prevent data leakage per TRIPOD + AI guidelines, all preprocessing, feature selection, and hyperparameter tuning were strictly confined to the training set, with the validation set reserved solely for final model assessment. We used Least Absolute Shrinkage and Selection Operator (LASSO) with 10-fold cross-validation to select predictors and determine the optimal λ value (λmin). The final predictors included in the model were identified through LASSO regression screening. Seven machine learning approaches were employed to predict anemia risk in intertrochanteric hip fracture patients: Logistic Regression (LR), Support Vector Machine (SVM), Decision Tree (DT), LightGBM, XGBoost, Artificial Neural Network (ANN), and Random Forest (RF). Optimal hyperparameters for each model were determined via grid search optimization [23]. This study utilized the training set for model development. Subsequently, model performance was evaluated using both the validation and test sets. The optimal predictive model was selected based on three criteria: clinical utility, calibration, and discriminative ability. Additionally, model performance was assessed using a confusion matrix, from which sensitivity, specificity, accuracy, and F1 score were calculated.

### 2.4. Machine Learning Algorithms

Using Python’s scikit-learn library, seven machine learning models [23,24,25,26] were constructed based on the selected feature variables: LR, SVM, DT, LightGBM, XGBoost, ANN, and RF. Optimal hyperparameters for each model were determined through grid search (5-fold cross-validation) to minimize overfitting. The best parameters for each model are as follows: DT (‘ccp_alpha’: 0.0, ‘max_depth’: 3, ‘max_features’: None, ‘min_samples_split’: 20); RF (n_estimators: 450, max_features: 2); SVM (‘C’: 0.1, ‘degree’: 2, ‘gamma’: ‘scale’, ‘kernel’: ‘linear’); XGBoost (‘learning_rate’: 0.1, ‘max_depth’: 3, ‘n_estimators’: 50, ‘subsample’: 0.8); LightGBM (‘colsample_bytree’: 1.0, ‘learning_rate’: 0.1, ‘n_estimators’: 100, ‘num_leaves’: 31, ‘subsample’: 0.6); ANN (‘activation’: ‘relu’, ‘hidden_layer_sizes’: (100)). Grid search-derived optimal hyperparameters effectively minimized overfitting risk and improved each model’s predictive performance on the training set.

### 2.5. SHAP Interpretability Analysis

The SHAP (Shapley Additive Explanations) method, rooted in game theory, enables the interpretation of outputs from any machine learning model [27]. By computing SHAP values, this approach quantified each predictor’s contribution to the model’s predictions. Higher SHAP values indicated positive impacts of variables on the output, whereas lower values denoted negative effects. We performed a comprehensive analysis of 15 variables and subsequently developed a user-friendly online application using Streamlit, enabling convenient access for surgeons.

### 2.6. Statistical Analysis

Continuous variables are expressed as median (interquartile range [IQR]) or mean ± standard deviation (range), with between-group comparisons conducted using the Student’s *t*-test or Wilcoxon rank-sum test. Categorical variables are presented as frequencies (percentages), and group differences were assessed using the chi-square test or Fisher’s exact test. A two-tailed *p*-value < 0.05 was deemed statistically significant. All statistical analyses were performed using Python 3.13.1 (Python Software Foundation, Wilmington, DE, USA), R 4.4.2 (The R Foundation for Statistical Computing, Vienna, Austria), and SPSS 29.0 (IBM, Armonk, NY, USA).

## 3. Results

### 3.1. Patient Characteristics

A total of 1125 patients were screened, of whom 310 were excluded (53 received conservative treatment, 217 had multiple fractures or severe trauma, 11 had pathological fractures, and 29 had preoperative severe anemia). The final cohort included 815 patients, with 761 allocated to the derivation set (70% training, 30% validation) and 54 allocated from January to June 2025 data for temporal validation. The temporal validation comprises 54 patients with 11 positive events (20.4% prevalence) and 43 negative events. Patient characteristics are summarized in Table 1. No significant differences were identified between the training and validation sets, indicating a relatively balanced distribution of variables across both. (Table 2).

### 3.2. Predictor Selection

**Figure 2:**
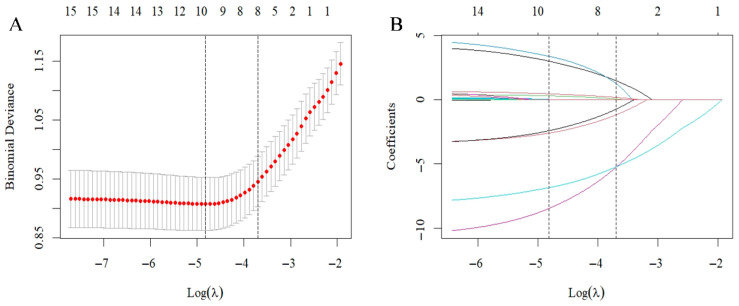
LASSO regression for feature selection. (**A**) Ten-fold cross-validation error curve. Left dashed line: lambda.min; right dashed line: lambda.1se. (**B**) Coefficient paths of features across log(lambda) values.

LASSO regression identified eight variables with an optimal lambda of 0.02469306, validated by 10-fold cross-validation. Figure 2 illustrates the LASSO regression feature selection process. Panel A shows binomial deviance versus Log(λ), where λ is the regularization parameter. The left dashed line indicates λ at minimum deviance; the right dashed line indicates optimal λ within one standard error. Panel B displays coefficient paths across λ values, with coefficients shrinking toward zero as λ increases. Numbers at top indicate non-zero coefficients retained at each λ. This analysis identified key predictive variables from the initial feature set for subsequent model development. The final selected features included: Type of anesthesia, diabetes, intraoperative transfusion volume, operation time, BMI, waiting time, preoperative hemoglobin level, and preoperative platelet count.

### 3.3. Model Development and Performance Comparison

**Figure 3:**
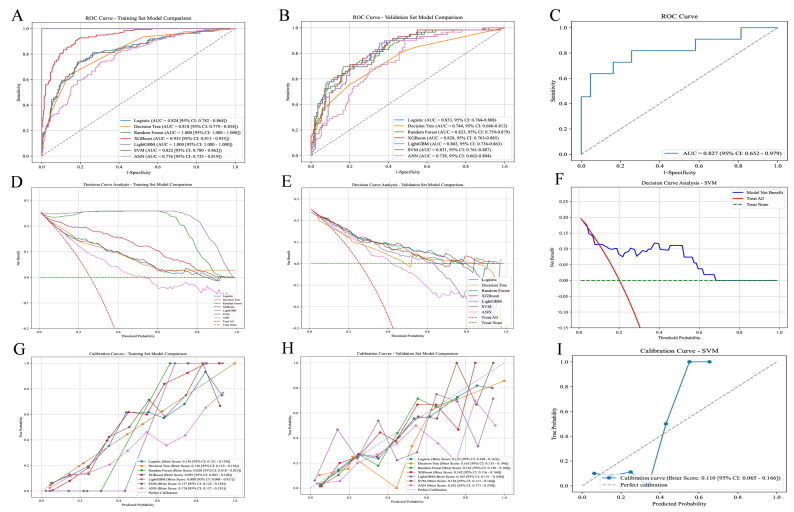
Performance evaluation of seven predictive models and comparison. (**A**) ROC Curves for the Training Set; (**B**) ROC Curves for the Validation Set; (**C**) ROC Curve for the SVM Model in the Test Set; (**D**) Decision Curve Analysis for the Training Set; (**E**) Decision Curve Analysis for the Validation Set; (**F**) Decision Curve Analysis for the SVM Model in the Test Set; (**G**) Calibration Curve for the Training Set; (**H**) Calibration Curve for the Validation Set; (**I**) Calibration Curve for the SVM Model in the Test Set.

Seven machine learning models were utilized to predict postoperative anemia in patients with intertrochanteric hip fractures. The performance of these models is presented in Figure 3, as illustrated by the receiver operating characteristic (ROC) curve, calibration curve, and decision curve analysis (DCA), providing a comprehensive evaluation of their discriminative ability, calibration, and clinical utility. By integrating every predictor across the training, validation, and test sets, SVM and LR demonstrated comparable discriminative performance. When forecasting anemia outcomes in all patients, the SVM model exhibited excellent discriminative capability in validation, with an AUC of 0.831 (95% CI 0.761–0.887), a sensitivity of 72.88%, a specificity of 68.05%, an accuracy of 69.30%, a precision of 44.33%, and an F1 score of 55.13%. The test set also showed strong performance, with an AUC of 0.827 (95% CI 0.652–0.979), an accuracy of 77.78%, a sensitivity of 72.73%, a precision of 47.06%, a specificity of 79.07%, and an F1 score of 57.14%. All model performance parameters are summarized in Table 3. The rationale for selecting SVM as the optimal model is as follows: SVM and LR exhibited comparable stability across datasets with minimal overfitting. Both achieved training AUC of 0.82 and validation AUC of 0.83, showing consistent discrimination capacity during model development. However, SVM outperformed LR on the independent test set with superior AUC (0.83 vs. 0.80) and markedly better sensitivity (0.7273 vs. 0.7272, with SVM maintaining this level more consistently). SVM’s F1 score (0.5714) further indicated better precision-recall balance than LR’s unweighted accuracy. In postoperative anemia prediction, even small AUC increments can translate to meaningful differences in patient outcomes when applied at scale. In conclusion, SVM was selected as the recommended model due to its optimal balance of stability, generalization, and discriminative performance on the independent test set.

### 3.4. Interpretability and Application of the Model

**Figure 4:**
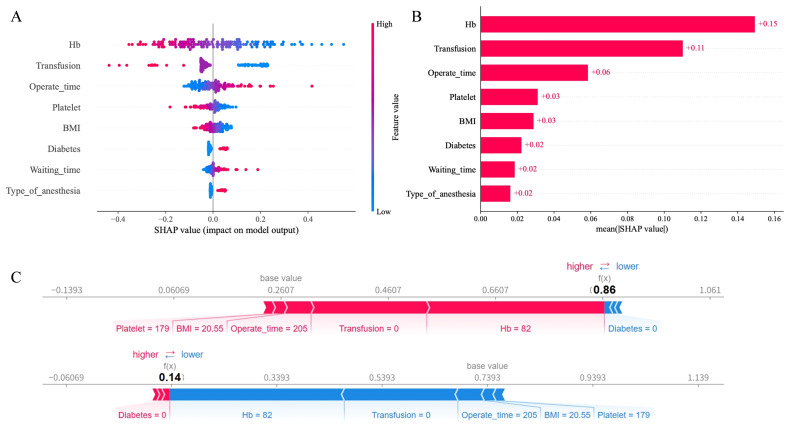
SHAP analysis of the SVM model. (**A**) Feature attribution plot. The *x*-axis represents SHAP values, with each dot indicating a feature instance. Red and blue colors denote higher and lower feature values, respectively. (**B**) Feature importance ranking based on mean absolute SHAP values. (**C**) Individual prediction explanations for two representative cases.

**Figure 5:**
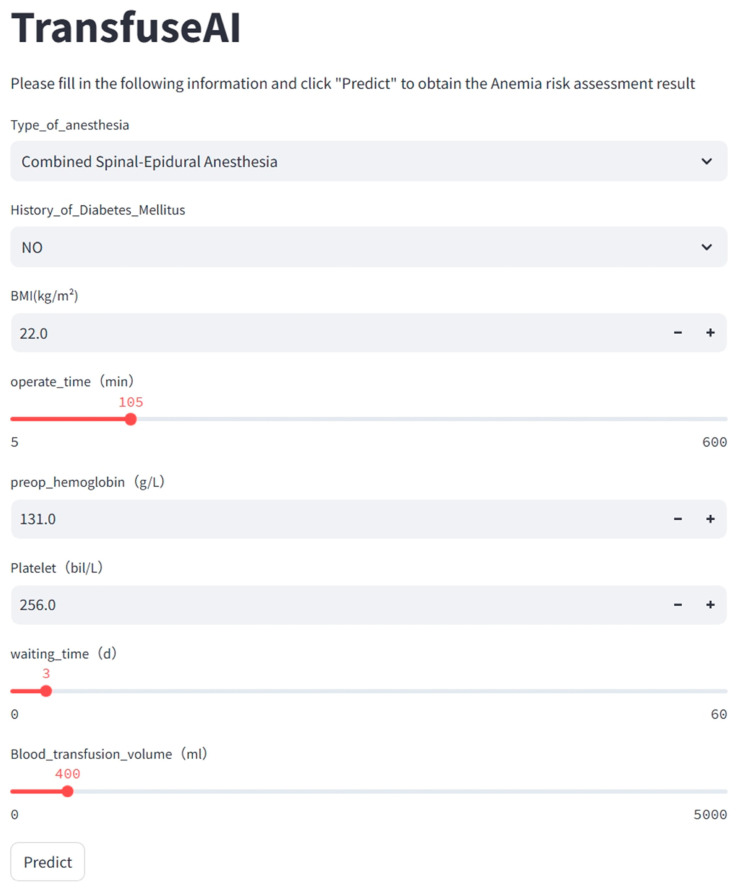
The application will automatically estimate the likelihood of postoperative anemia in intertrochanteric hip fracture patients.

Figure 4A shows the SHAP swarm plot for interpreting the SVM model predictions. The plot quantifies the contribution of each feature toward the outcome of postoperative anemia. The *X*-axis (SHAP value) denotes the direction and magnitude of each feature’s effect: a higher SHAP value corresponds to an increased risk of anemia, whereas a lower value indicates a reduced risk. The color gradient from red to blue represents feature magnitude, with red indicating high values and blue indicating low values. Preoperative hemoglobin and intraoperative transfusion volume exerted the most pronounced effects on anemia prediction. Higher preoperative hemoglobin, greater intraoperative transfusion volume, and shorter operation time were associated with decreased anemia risk. In contrast, diabetes, lower BMI, lower platelet count, longer preoperative waiting time, and general anesthesia were linked to an elevated risk of postoperative anemia. Figure 4B presents the variable importance ranking, where preoperative hemoglobin, intraoperative transfusion volume, and operation time were identified as the top three influential features. We further developed and deployed a user-friendly online prediction platform using Streamlit (https://transfuseai.streamlit.app/ (accessed on 20 February 2026)), with the source code deposited in GitHub, as illustrated in Figure 5. This web-based tool enables clinicians to conveniently estimate postoperative anemia risk in patients with intertrochanteric hip fractures by inputting key clinical variables into the designated interface, allowing efficient and individualized risk assessment. For example, a patient with no history of diabetes, a BMI of 22 kg/m^2^, who underwent combined spinal-epidural anesthesia, had a preoperative hemoglobin of 131 g/L, preoperative platelets of 256 bil/L, a preoperative waiting time of 3 days, and received 400 mL of suspended red blood cells during surgery, has an approximately 1.53% probability of developing hemoglobin levels below 80 g/L within 7 days postoperatively.

## 4. Discussion

This study utilized retrospective clinical data from Tangdu Hospital and multiple machine learning models to develop prediction models for postoperative anemia risk in intertrochanteric hip fracture patients. Due to the scarcity of reliable and evidence-based tools, decisions regarding intraoperative blood transfusion remain largely subjective, relying on clinical judgment. To address this gap, we developed predictive tools specifically tailored to this patient population, incorporating standardized variables including demographics, medical history, and perioperative health-related factors. A key strength of this study is the use of real-world hospital data integrated with machine learning to identify major risk factors for postoperative anemia. Our validated predictive model enables accurate risk stratification, assisting surgeons in developing individualized perioperative blood management strategies. This approach primarily aims to predict and prevent postoperative anemia, and the optimized perioperative blood management based on anemia risk stratification may indirectly contribute to more rational intraoperative blood transfusion decisions.

Via LASSO regression, we screened eight critical predictive variables. As shown in Figure 4, elevated preoperative hemoglobin, platelet count and BMI were associated with lower anemia risk. In contrast, prolonged operation time, extended waiting time, general anesthesia and diabetes significantly increased anemia incidence. These associations have been widely reported in previous clinical studies. The identified risk factors align with recognized pathophysiological mechanisms. Preoperative hemoglobin emerges as the dominant predictor, where each 1 g/L decrement increases anemia risk by 5%—a dose–response relationship reflecting the narrow physiological reserve in elderly trauma patients [28]. The increased risk of anemia associated with general anesthesia corroborates Previous studies [29,30] and likely reflects differential vasomotor regulation and erythropoietic suppression compared to combined spinal-epidural anesthesia. We selected operation time as a predictor variable, as it is objectively documented in anesthesia records with triple-verification (surgeon, anesthesiologist, and nurse), reliably reflecting surgical trauma magnitude. Operation time and preoperative waiting days capture the cumulative physiological stress of delayed fixation and prolonged surgical exposure, reinforcing the imperative for expeditious surgery in high-risk patients [31,32]. A lower BMI often suggests underlying malnutrition or reduced muscle mass. Such patients may present with lower preoperative hemoglobin levels and possess a diminished capacity to compensate for surgical blood loss [33,34]. Furthermore, patients with low BMI are frequently associated with insufficient protein reserves, which can impair the synthesis of coagulation factors and the integrity of blood vessel walls, potentially increasing perioperative blood loss. Although not directly causal, the association between preoperative platelet count and postoperative anemia is well-established as an independent predictor by numerous evidence-based studies [35]. There is a medical consensus that perioperative management should focus on correcting platelet abnormalities to mitigate postoperative complications, including anemia, which corroborates our findings [36]. Diabetic patients have a significantly higher risk of developing postoperative anemia compared to non-diabetic patients. Multiple studies [37] have shown that diabetes is not only an independent risk factor for anemia but may also exacerbate the negative impact of anemia on postoperative prognosis, which is consistent with the findings of our study. For diabetic patients, it is recommended to actively correct anemia before surgery, assess renal function and nutritional status (such as iron, folate, and vitamin B12 levels), and optimize glycemic control [38]. Hemoglobin levels should be closely monitored during the perioperative period.

Our results demonstrate that SVM surpasses the other six models in accuracy, sensitivity and F1 score in the validation set. It also achieved favorable specificity, accuracy and AUC in the independent test set. SVM exhibits strong performance in validation and test set, with AUC values of 0.831 (95% CI 0.761–0.887) and 0.827 (95% CI 0.652–0.979), respectively, indicating exceptional predictive accuracy for positive samples. According to the DCA of the validation and test set, interventions guided by the prediction model tool yielded outstanding results (Figure 3E,F). Furthermore, the calibration curve was used to evaluate model calibration, with SVM achieving a Brier score of 0.136 (95% CI 0.113–0.164) in the validation set and 0.11 (95% CI 0.065–0.166) in the test set, demonstrating strong calibration and predictive accuracy. In conclusion, SVM shows exceptional generalizability and stability across diverse populations, leading us to choose it as the best predictive tool.

Previous studies on anemia risk prediction in intertrochanteric fracture patients have explored the utility of traditional predictive models. Bian et al. conducted a study involving 576 hip fracture patients from a single institution and reported that their multivariable LR model exhibited excellent predictive performance for postoperative blood transfusion [11]. Similarly, Wang et al. conducted a study involving 119 patients with intertrochanteric hip fracture and employed LR to establish a simplified model for predicting postoperative blood transfusion [39]. These studies offer significant insights into predicting postoperative blood transfusion risk in hip fracture patients through the construction of traditional models. However, our study offers several distinct advantages: (1) Our study utilized postoperative hemoglobin level as the predictive outcome, which offers a more objective measure compared to the endpoints used in prior research. Notably, predicting postoperative anemia and guiding intraoperative blood transfusion are two distinct clinical concepts: the indication for blood transfusion lacks a unified standard and varies considerably across hospitals and surgeons, which limits the generalizability of predictive models focused on transfusion. In contrast, predicting postoperative anemia provides a direct, objective basis for intraoperative blood management (e.g., targeted correction of preoperative and intraoperative risk factors), which is independent of transfusion decision-making and focuses on improving the patient’s underlying physiological status to prevent anemia. (2) The present study enrolled 815 patients for modeling, exceeding the sample sizes of prior conventional prediction models. (3) We constructed an anemia prediction model using seven machine learning algorithms. These models can capture key features and complex nonlinear associations, frequently yielding better predictive performance than conventional regression methods. (4) This study also included 54 patients for temporal validation, further confirming the robustness and generalization of our prediction tool. (5) Most importantly, we constructed a user-friendly online clinical decision tool that allows clinicians to identify patients at high risk of postoperative anemia conveniently at any time, supporting early identification and prompt intervention. These innovations have not been reported in prior research.

The SVM-based online prediction tool developed in this study holds significant clinical implications for the prevention and management of postoperative anemia. During the intraoperative period, healthcare professionals can rapidly obtain individualized anemia risk probabilities at the completion of surgery through this online platform, enabling real-time adjustments to postoperative monitoring and care protocols. Once high-risk patients are identified, targeted perioperative blood management strategies (e.g., optimizing preoperative hemoglobin levels, correcting nutritional deficiencies) can be initiated to prevent postoperative anemia. The core value of this tool lies in its ability to accurately predict anemia occurrence and guide clinical decision-making related to anemia prevention through adjustable risk factor thresholds. This tool is designed for postoperative anemia prediction and provides a favorable basis for more rational transfusion decisions.

This approach focuses on the prevention of postoperative anemia, which in turn may help reduce the need for blood transfusion in some cases and indirectly contribute to the conservation of valuable blood resources and the avoidance of potential transfusion-related complications. Amid current blood resource constraints, this anemia prediction model significantly improves the efficiency of perioperative anemia management, enabling physicians to focus targeted interventions on high-risk patients and achieve scientific, refined management of anemia prevention. Through this clinical decision support tool for anemia prediction, future efforts can further advance the intelligent development of perioperative blood management, ultimately improving overall outcomes and quality of life for elderly hip fracture patients.

In this study, our machine learning models, especially the SVM model, exhibited promising performance in predicting postoperative anemia in this large-scale dataset. Nonetheless, several limitations should be acknowledged. First, although our database was of high quality and the temporal validation showed acceptable results, the model was developed retrospectively based on a single-center dataset, which may inherently introduce selection and measurement biases during data acquisition. Second, as a retrospective cohort study, our research lacked long-term longitudinal follow-up data required for dynamic evaluation of anemia trajectories over time, in contrast to prospective investigations. Moreover, temporal variations in hospital management protocols and regional healthcare policies may potentially affect model generalizability when applied to current clinical populations. Thirdly, the temporal validation set (n = 54) is insufficient to support strong claims of the model’s generalizability, which is a critical limitation that must be clearly acknowledged. Finally. RF and LightGBM achieved perfect discrimination on the training set (AUC = 1.00, accuracy = 1.00), which strongly suggests overfitting to the training data. This is further corroborated by their substantial performance decay on the test set: Random Forest’s AUC dropped by 22% and LightGBM’s by 23%. Such dramatic training–test discrepancies indicate that these models memorized idiosyncratic patterns in the training data rather than learning generalizable relationships. XGBoost exhibited similar though less severe overfitting (training AUC 0.94, test AUC 0.80, 14% decay). These models were deliberately excluded from final selection despite their impressive training metrics, as clinical utility requires generalization to new patients—a criterion that outweighs in-sample performance. This conservative approach aligns with machine learning best practices for healthcare applications, where model robustness on unseen data is paramount. Future studies should prioritize external validation with contemporary datasets to further verify the model’s stability and clinical applicability over time.

## 5. Conclusions

In summary, this study developed and validated seven machine learning models using retrospective data from Tangdu Hospital to predict postoperative anemia in intertrochanteric hip fracture patients. The SVM model outperformed six comparator algorithms, achieving optimal discrimination (test AUC = 0.83) and clinically actionable sensitivity (72.73%) after threshold optimization. Key predictors, including type of anesthesia, diabetes, intraoperative transfusion volume, operation time, BMI, waiting time, preoperative hemoglobin level, preoperative platelet count, and intraoperative blood loss, enable targeted preventive interventions and resource allocation. Limitations include a single-center design, modest sample size, and the need for extended temporal validation. Future work will focus on multicenter prospective implementation and integration with electronic health record systems. This interpretable, scalable prediction tool advances precision perioperative therapy for this high-risk population.

## Figures and Tables

**Table 1:** Comparison of baseline data between anemia group and non-anemia group.

Characteristics	Total (N = 815)	Non-Anemia (N = 607)	Anemia (N = 208)	*p*
Age, Median (IQR)	76 (67, 83)	75 (66, 82)	77 (69, 84)	0.027
BMI, Median (IQR)	22.5 (20.0, 24.3)	22.5 (20.2, 24.5)	21.7 (18.7, 24.1)	<0.001
Hb, Median (IQR)	105 (93, 118)	110 (98, 121)	94 (86, 104)	<0.001
Albumin, Mean ± SD	35.02 ± 3.88	35.37 ± 3.88	34.01 ± 3.70	<0.001
Calcium, Median (IQR)	2.13 (2.04, 2.20)	2.13 (2.05, 2.20)	2.10 (2.02, 2.19)	0.012
Platelet, Median (IQR)	196 (152, 261)	197 (155, 263)	187 (146, 247)	0.023
Operation time, Median (IQR)	145 (100, 196)	140 (100, 190)	160 (108, 220)	0.004
Waiting time, Median (IQR)	8 (6, 11)	8 (6, 10)	8 (6, 12)	0.305
Transfusion, Median (IQR)	400 (0, 400)	400 (0, 400)	400 (0, 400)	0.003
Gender, n (%)				0.297
Male	380 (46.6)	290 (47.8)	90 (43.3)	
Female	435 (53.4)	317 (52.2)	118 (56.7)	
Evans, n (%)				0.06
Type 1	31 (3.8)	25 (4.1)	6 (2.9)	
Type 2	194 (23.8)	156 (25.7)	38 (18.2)	
Type 3	89 (10.9)	71 (11.7)	18 (8.7)	
Type 4	422 (51.8)	300 (49.4)	122 (58.7)	
Type 5	79 (9.7)	55 (9.1)	24 (11.5)	
Diabetes, n (%)				0.205
No	620 (76.1)	469 (77.3)	151 (72.6)	
Yes	195 (23.9)	138 (22.7)	57 (27.4)	
Osteoporosis, n (%)				0.253
No	339 (41.6)	260 (42.8)	79 (38.0)	
Yes	476 (58.4)	347 (57.2)	129 (62.0)	
Hypertension, n (%)				0.931
No	439 (53.9)	328 (54.0)	111 (53.4)	
Yes	376 (46.1)	279 (46.0)	97 (46.6)	
Type of anesthesia, n (%)				0.009
CSEA	649 (79.6)	497 (81.9)	152 (73.1)	
GA	166 (20.4)	110 (18.1)	56 (26.9)	

**Table 2:** Comparison of baseline data between Training and Validation sets.

Characteristics	Training Set (N = 533)	Validation Set (N = 228)	*p*
Age, Mean ± SD	73 ± 12	72 ± 14	0.5312
BMI, Mean ± SD	22.39 ± 3.74	22.19 ± 3.23	0.4294
Hb, Mean ± SD	107 ± 18	107 ± 18	0.8519
Albumin, Mean ± SD	35.15 ± 3.83	34.93 ± 3.95	0.5456
Platelet, Mean ± SD	212 ± 98	224 ± 94	0.1228
Calcium, Mean ± SD	2.12 ± 0.14	2.12 ± 0.13	0.7751
Operation time, Mean ± SD	161 ± 81	174 ± 105	0.3461
Waiting time, Mean ± SD	9 ± 7	10 ± 6	0.0663
Transfusion, Median (IQR)	400 (0, 400)	400 (0, 400)	0.5312
Gender, n (%)			0.732
Male	252 (47.3)	104 (45.6)	
Female	281 (52.7)	124 (54.4)	
Hypertension, n (%)			0.2064
No	278 (52.2)	131 (57.5)	
Yes	255 (47.8)	97 (42.5)	
Diabetes, n (%)			0.8331
No	406 (76.2)	176 (77.2)	
Yes	127 (23.8)	52 (22.8)	
Osteoporosis, n (%)			0.2404
No	217 (40.7)	104 (45.6)	
Yes	316 (59.3)	124 (54.4)	
Type of anesthesia, n (%)			0.1653
CSEA	432 (81.1)	174 (76.3)	
GA	101 (18.9)	54 (23.7)	
Evans, n (%)			0.5788
Type 1	19 (3.6)	12 (5.3)	
Type 2	118 (22.1)	59 (25.9)	
Type 3	65 (12.2)	24 (10.5)	
Type 4	281 (52.7)	112 (49.1)	
Type 5	50 (9.4)	21 (9.2)	

**Table 3:** Detailed performance metrics of seven machine learning models.

		LR	DT	RF	XGBoost	LightGBM	SVM	ANN
training	AUC	0.82	0.82	1.00	0.94	1.00	0.82	0.78
	Accuracy	75.80%	79.92%	100.00%	84.62%	99.81%	74.67%	58.35%
	Precision	52.24%	60.69%	100.00%	64.43%	99.28%	50.71%	36.96%
	Sensitivity	76.09%	63.77%	100.00%	90.58%	100.00%	77.54%	86.23%
	Specificity	75.70%	85.57%	100.00%	82.53%	99.75%	73.67%	48.61%
	F1 Score	61.95%	62.19%	100.00%	75.30%	99.64%	61.32%	51.74%
validation	AUC	0.83	0.74	0.82	0.83	0.80	0.83	0.74
	Accuracy	70.61%	73.25%	68.42%	73.68%	73.25%	69.30%	58.33%
	Precision	45.83%	48.53%	43.93%	49.41%	48.84%	44.33%	37.32%
	Sensitivity	74.58%	55.93%	79.66%	71.19%	71.19%	72.88%	89.83%
	Specificity	69.23%	79.29%	64.50%	74.56%	73.96%	68.05%	47.34%
	F1 Score	56.77%	51.97%	56.63%	58.33%	57.93%	55.13%	52.74%
test	AUC	0.80	0.72	0.78	0.80	0.77	0.83	0.79
	Accuracy	81.48%	81.48%	72.22%	81.48%	79.63%	77.78%	57.41%
	Precision	53.33%	66.67%	40.00%	53.85%	50.00%	47.06%	31.25%
	Sensitivity	72.72%	18.19%	72.73%	63.63%	45.45%	72.73%	90.91%
	Specificity	83.72%	97.67%	72.10%	86.04%	88.37%	79.07%	48.84%
	F1 Score	61.53%	28.57%	51.61%	58.33%	47.62%	57.14%	46.51%

## Data Availability

The datasets used and/or analyzed during the current study are available from the corresponding authors on reasonable request. The data are not publicly available due to [patient privacy and ethical confidentiality requirements].
